# Velcro-Like System Used to Fix a Protective Faecal Shield on Weevil Larvae

**DOI:** 10.1371/journal.pone.0170800

**Published:** 2017-01-26

**Authors:** Jiří Skuhrovec, Robert Stejskal, Filip Trnka, Andrea di Giulio

**Affiliations:** 1 Group Function of Invertebrate and Plant Biodiversity in Agro-Ecosystems, Crop Research Institute, Praha 6 – Ruzyně, Czech Republic; 2 Administration of Podyji National Park, Znojmo, Czech Republic; 3 Department of Ecology & Environmental Sciences, Faculty of Science, Palacký University Olomouc, Olomouc, Czech Republic; 4 Department of Science, University Roma Tre, Rome, Italy; University of Innsbruck, AUSTRIA

## Abstract

The last instar larva and pupa of *Eucoeliodes mirabilis* (A. Villa & G. B. Villa, 1835) (Curculionidae: Ceutorhynchini) are described using drawings and SEM images and are compared and keyed with already described larvae of 58 other ceutorhynchinae taxa. The larval body has an effective combination of morphological adaptations that assist a unique biological defensive strategy. All larval stages of *E*. *mirabilis* feed ectophytically on leaves of *Euonymus europaeus* L. (Celastraceae), and the larval body is covered with a thick faecal shield. The fixation of this protective shield on the larval back is performed by a peculiar dorsal microsculpture composed of a dense carpet of microtrichia on the thorax and abdomen, which serves effectively as a velcro system. Because of this strategy, macrosetae on the larval and pupal body of *E*. *mirabilis* are completely reduced. Larvae of *E*. *mirabilis* also have distinct morphological adaptations for protecting the spiracles against intrusion of faeces and avoiding occlusion of the tracheal system: a) microtrichia around spiracles are slightly shorter, distinctly stronger and are arranged with high-density and in clusters and b) spiracles are protected by an external safety valve. This strategy of *E*. *mirabilis* larvae is unique, although somewhat similar to that of Criocerinae and Blepharida-group leave beetles (Galerucinae) (both Coleoptera: Chrysomelidae), but with distinctly different morphological adaptations.

## Introduction

The endless adaptations and counter-adaptations among three trophic levels (plants, herbivores and their enemies) are responsible for the amazing variety of defensive strategies recorded for both plants and their herbivorous insects [1]. To avoid the attacks of predators and parasitoids, herbivorous insects have developed many types of defensive strategies that vary from avoiding detection using visual camouflage to deceiving enemies by mimicking unpalatable species [2]. One of the most astonishing defensive strategies may be the faecal ecology of insects, which has received some attention [3]. Immature stages of five clades of leaf beetles, Cassidinae, Criocerinae, Cryptocephalinae, Lamprosomatinae and Galerucinae, use faeces as a material for building domiciles and protective shields/coats against their enemies [4–20]. The shields provide a mechanical defence and may also include chemicals that originated from the host plant or derivatives of these compounds produced by the larvae [21–23].

Each of these clades of leaf beetles shows slight differences in the use of faeces. The strategy of using faeces as a shield is most recognized for the larvae of tortoise beetles, Cassidinae [15]. Eisner and his colleagues [4] first reported that the faecal shields of tortoise beetle larvae are the primary defensive strategy against predators. Some larvae of Cassidinae have special super-anal processes to carry the defensive shields, which are composed of shed larval skins (exuviae) and faeces that are often retained after pupation [15]. These larvae can move this defensive shield to the side, in contrast to the remaining four groups (Criocerinae, Cryptocephalinae, Lamprosomatinae and Galerucinae), which have faeces only on the dorsum and cannot move the defensive shield from side to side. Larvae in the clade Lamprosomatinae and Cryptocephalinae, sister taxa referred to as Camptosomata, are defined by a bell-shaped case from faeces and plant material as portable enclosures, but the head and legs are not protected during walking [16, 17]. Larval chaetotaxy of both these groups is reduced [16, 17]. In Galerucinae, only larvae of four genera, *Blepharida* Chevrolat, *Diamphidia* Gerstaecker, *Podontia* Dalman, and *Polyclada* Chevrolat, use faecal coats [13, 24]. All larval instars partially or completely cover the terga with faeces, resembling semirigid pellets or a wet mass. Larvae of Criocerinae have the anus oriented vertically, and then digestive wastes (faeces) accumulate on the dorsum to partially or entirely cover the larvae [18]. A faecal shield acts primarily as a physical and chemical barrier with deterrent metabolites that include fatty acids (e.g., octadecanoic acid and phytol). Larvae continuously form this protective shield after each moulting, and the dorsal vesture consists of short, relatively sparse setae [8, 18, 25].

During biological investigations on the weevil *Euceliodes mirabilis* (A. & G. B. Villa, 1835), we observed some protective behaviours of the larvae that included an ectophytic habitat and the use of a faecal shield. To date, this peculiar defensive strategy is known only in the family Chrysomelidae. *Eucoeliodes* Smreczyński, 1974 is a rare, monotypic genus [26] formerly regarded as a subgenus within *Coeliodes* Schoenherr, 1837 [27, 28], and now considered as genus related to the genus *Coeliodes* and other relative genera as e.g. *Neocoeliodes* Colonnelli, 1984 [29]. The distribution of *E*. *mirabilis* includes Central Europe (Austria, Czech Republic, Hungary, Slovakia and Switzerland) and a part of southern Europe (Croatia, Italy and Slovenia) [26]. The information on the biology of this weevil is very poor, although reports are available that the weevil is collected on European spindle (*Euonymus europaeus* L., Celastraceae) [27–30] and alder buckthorn (*Frangula alnus* Mill., Rhamnaceae) [31]. Numerous populations of *E*. *mirabilis* were found in two localities in southern Moravia (Czech Republic) at the northernmost edge of the range of the species. In this paper, we present new biological and morphological data on the larval development and behaviour of the weevil *Eucoeliodes mirabilis*.

## Materials and Methods

### Insect collection and laboratory breeding

The material used to describe immature stages of *Eucoeliodes mirabilis* was collected, and field observations were conducted in two study areas: 1) Czech Republic, Moravia mer., National Park Podyjí, Znojmo env., 250 m a.s.l., visit dates: 19.v.2010, 2 ex.; 29.iv.2012, ca 30 ex. (adults); 10.v.2012, ca 20 ex. (only observed, adults and larvae), leg. Robert Stejskal (RS); 19.v.2012, more than 100 larvae (only observed on three bushes), leg. RS; 30.v.2014, more than 100 larvae (only observed on three bushes)–ca 10 ex. collected for rearing, leg. RS; 7.vi.2014, 2 adults and ca 20 larvae (only observed on three bushes), leg. RS and 2) Czech Republic, Moravia centr., Boskovice env., 350 m a. s. l., visit dates: 28.vii.2012 (old feeding marks on leaves), 23.v.2015, ca 30 adults (7 collected) and ca 100 small larvae (observed on ca 10 bushes)–ca 20 larvae collected for rearing, leg. RS. Descriptions of immature stages were performed on ten larvae and five pupae.

**Fig 1:**
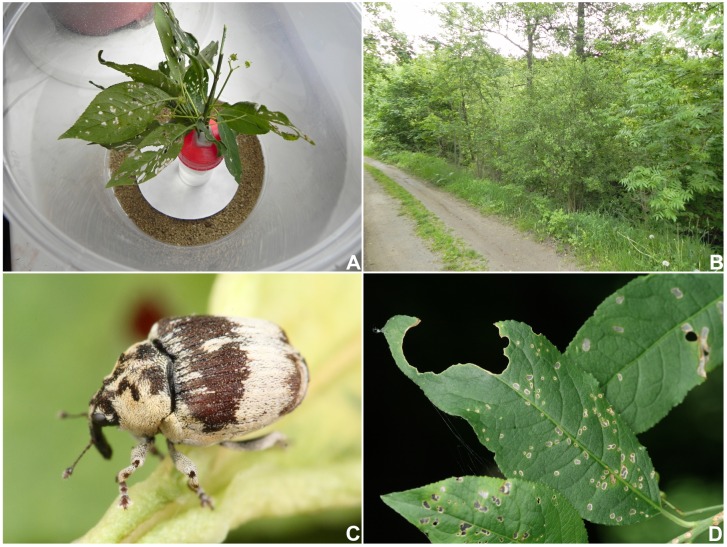
*Eucoeliodes mirabilis*: adult, habitat and host plant. (A) Larvae rearing in a plastic container with a few branches of *Euonymus europaeus*. (B) Habitat in the Boskovice (Czech Republic). (C) Adult. (D) Feeding marks of adults. Photos A and B by R. Stejskal, and C and D by F. Trnka.

Laboratory observations were conducted in Znojmo, Czech Republic. Adults and larvae were placed in a covered small plastic container with a few branches of *Euonymus europaeus* (Fig 1A). Fine sand obtained in a pet shop was placed at the bottom of the container. The container containing the host plants and larvae was placed near the window at room temperature and slightly moistened approximately once every 3–5 days.

To define the concepts of endo/ectophyty and endo/ectophagy, we followed Oberprieler et al. [32]: endophyty refers to living inside plant tissues, whereas endophagy denotes feeding inside any substrate (e.g., soil, water). Similarly, ectophagous species feed and live on the surface of a host, and when the host is a plant, the species are ectophytic.

### Morphological descriptions

Part of the larval and pupal material was preserved in Pampel fixation liquid (4 parts glacial acetic acid, 6 parts 4% formaldehyde, 15 parts 95% ethanol and 30 parts distilled water) and used for the morphological descriptions. These specimens are now deposited in the Group Function of Invertebrate and Plant Biodiversity in Agro-Ecosystems of the Crop Research Institute (Prague, Czech Republic). The collectors identified the plants. To prepare the slides, we followed May [33]: a larva was decapitated, and the head was cleared in a 10% potassium hydroxide (KOH) solution and then rinsed in distilled water. After clearing, the mouthparts were separated from the head capsule, and the head capsule and all mouthparts were mounted on permanent microscope slides in Euparal. All other body parts were mounted on temporary microscope slides in 10% glycerine.

The observations and measurements were conducted using a light microscope with calibrated oculars (Olympus BX 40 and Nikon Eclipse 80i). The following characteristics were measured for each larva: head width, length of the body (larvae fixed in a C-shape were measured in segments), and width of the body in the widest place (i.e., metathorax or abdominal segments I–IV). For the pupa, the length and the width at the widest place were measured. The thorax and abdomen were not sclerotised, and it is unlikely that the fixation process altered the proportions of the weevils; measurements of these parts are provided for comparison purposes only.

Drawings were created with a drawing tube on a light microscope and edited using programs as Adobe Photoshop 10, Corel Photo-Paint 11, and GIMP 2. The thoracic spiracle is located on the prothorax near the boundary of the prothorax and mesothorax, as shown in the drawing (see Morphology of mature larva), but this spiracle is of mesothoracic origin [34, 35]. Drawings of body illustrate the thoracic and abdominal spiracles; the numbers of setae of the bilateral structures are given for one side.

We used the terms and abbreviations for the setae of the mature larva and pupa found in Scherf [36], May [33, 37] and Marvaldi [38, 39].

### Scanning electron microscopy

To clean the specimens, larvae preserved in EtOH 70% were gradually re-hydrated to distilled water, maintained overnight in oven at 45°C in a detergent-water solution (10% Svelto^®^ dishwashing liquid in distilled water), cleaned manually with a thin brush, and rinsed 3 times in distilled water. Then, the specimens were dehydrated by passing through a graded ethanol series, critical point-dried in a Balzer Union CPD 030 unit, gold coated in an Emitech K550 unit, and finally examined using the field emission SEM column of a Dualbeam (FIB/SEM) Helios Nanolab (FEI Company, Eindhoven, The Netherlands) at the L.I.M.E. (Roma Tre University, Rome, Italy), with secondary electrons (SE) and an operating voltage of 5 kV.

## Results

### *Eucoeliodes* (s.str.) *mirabilis* (A. & G. B. Villa, 1835)

#### Biological notes

**Habitat**: In both study areas (Znojmo env., Boskovice env.), the weevil occurred in mixed broadleaved forests near watercourses. Tree/shrub layer: *Fraxinus excelsior* L. (Oleaceae), *Alnus glutinosa* (L.) Gaertn. (Betulaceae), *Robinia pseudoacacia* L. (Fabaceae), *Acer campestre* L. (Sapindaceae), *Ulmus laevis* Pall. (Ulmaceae), *Euonymus europaeus* L. (Celastraceae), and *Sambucus nigra* L. (Adoxaceae). Herb layer: *Aegopodium podagraria* L. (Apiaceae), *Lamium maculatum* L. (Lamiaceae), *Galium aparine* L. (Rubiaceae), *Chelidonium majus* L. (Papaveraceae), *Impatiens parviflora* DC. (Balsaminaceae), and *Urtica dioica* L. (Urticaceae). The weevil preferred partly shadowed shrubs growing on forest edges (e.g., along roads and meadow edges, among others) (Fig 1B). Of note, although *Euonymus europaeus* was widely distributed in the study areas, the weevil occurred locally.

**Adult activity**: Adults (Fig 1C) appeared on the host plants at the end of April and immediately began to feed on leaves and twigs, where small holes were chewed (Fig 1D). Mating couples were observed from the end of April to the beginning of May. The last adults were observed to the beginning of June. In our database, we have records of adult *E*. *mirabilis* from other localities from the second half of June, which might indicate a new generation. No collecting data from summer and autumn months were available for the study areas or in other localities and published sources. The weevils may overwinter as adults.

**Fig 2:**
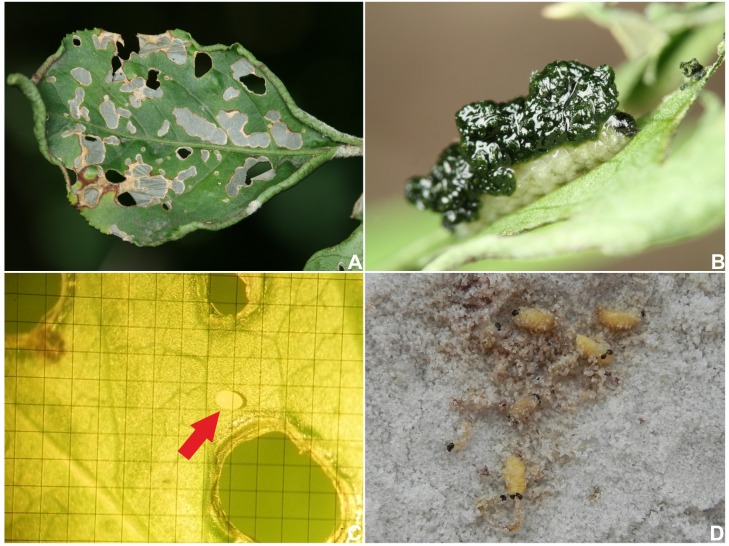
*Eucoeliodes mirabilis*: adult, host plant and larvae. (A) Feeding marks of larvae. (B) “Faecal shield” on larva. (C) An egg on a leaf of *Euonymus europaeus*. (D) Free pupae without any additional protection in the laboratory conditions. Photos A and B by F. Trnka; and C and D by R. Stejskal.

**Larval development**: Larvae were observed from the 10^th^ May to mid-June. The lower surface of a leaf was the site for larval feeding (Fig 2A). The larval body was covered with a “faecal shield”–viscid mucus and a dark substance reminiscent of excrements (Fig 2B). Young larvae resembled, at a glance, a small bird dropping and therefore could be easily overlooked. Depending on the age of the larva, feeding marks left patterns of different sizes and shapes on a leaf (Fig 2A and 2B). Initially, the feeding of young larvae is only superficial, and the epidermis remains largely untouched, whereas the feeding of older larvae leaves small holes or “windows” on the leaf (Fig 2A). A leaf can host up to 2 larvae. Feeding marks are very conspicuous; therefore, occupied host plants are easy to identify in the field, particularly at the end of May when the last larval instars feed intensively. We found no pupae in the field either on the host plants or in the litter on the ground.

**Rearing experiments**: Adults in captivity (ca 20 specimens) fed readily on host plants, mated, and laid only a total of 3 eggs, which dried out. Mating and oviposition were observed at the beginning of May 2012. The eggs were found on the lower surface of a leaf and were oval and lightly-colored, ca. 1.1 mm in length and ca. 0.7 mm in width (Fig 2C). The success in rearing the larvae, collected from the host plants in the field, was limited. Soon after placement on leaves in the rearing container, the larvae lost the protective shield, fed reluctantly, and moved around chaotically. Most specimens withered day by day and died by the end of the study. Pupation was observed in a few specimens (N = 4), and the larvae pupated on the surface of the sand that covered the bottom of the rearing dish. The pupae were free, without any additional protection, e.g., cocoon or earthen cell (Fig 2D). For larvae collected on the 23^rd^ May, the pupation date was between the 3^rd^ and 6^th^ June.

#### Morphology of mature larva

**Measurements (in mm)**: Body length: 6.0–7.0 (mean: 6.5). The widest place in the body (abdominal segments II–V) measured up to 2.0. Head width: 0.6–0.7 (mean: 0.6).

**Colouration**: Black or dark brown head (Fig 2B). All thoracic and abdominal segments white (Fig 2B and 2D); only dorsum of pronotum with elongated, light brown stripe.

**Fig 3:**
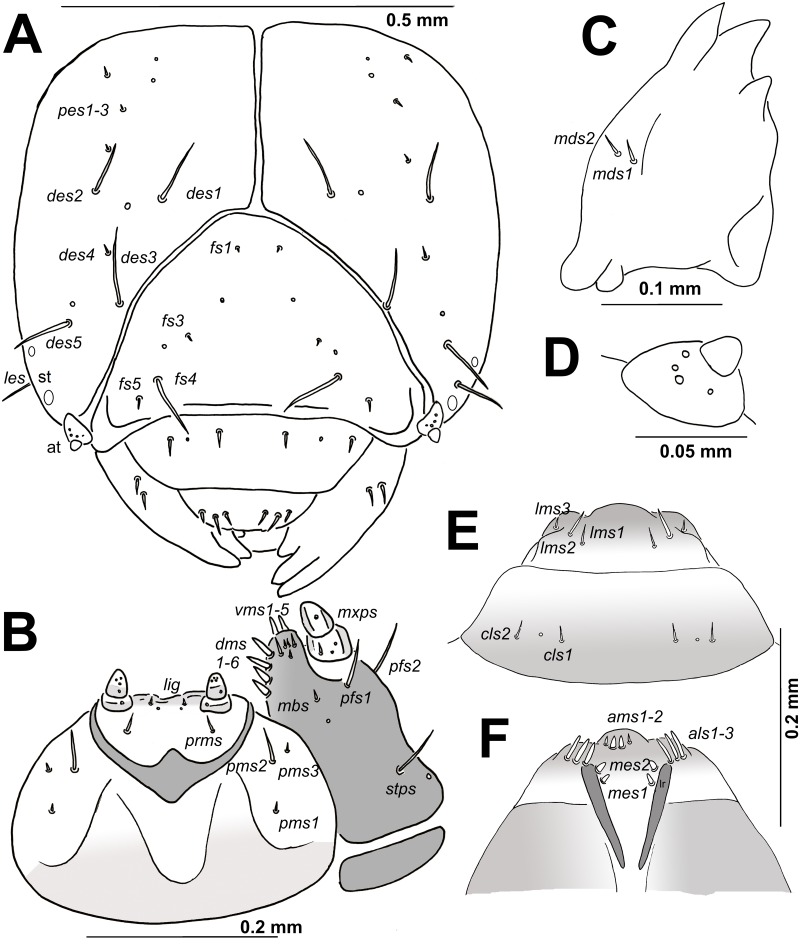
*Eucoeliodes mirabilis*: head and mouthparts of mature larva. (A) Anterior view of head. (B) Labium with left maxilla, ventral view. (C) Right mandible. (D) Antenna. (E) Labrum and clypeus. (F) Epipharynx. Abbreviations: *des*–dorsoepicranial seta(e), *fs*–frontal s., *les*–lateroepicranial s., *ves*–ventroepicranial s., at—antenna, st—stemmata, *cls*–clypeal s., *lms*–labral s., *ams*–anteromedial s., *als*–anterolateral s., lr—labral rods, *mds*–mandible dorsal s., *dms*–dorsal malae s., *vms*–ventral malae s., *mxps*–maxillary palps s., *pfs*–palpiferal s., *stps*–stipal s., *mbs*–mandible basiventral s., *prms*–prelabial s., *pms*–postlabial s., and *ligs*–ligular s.

**Fig 4:**
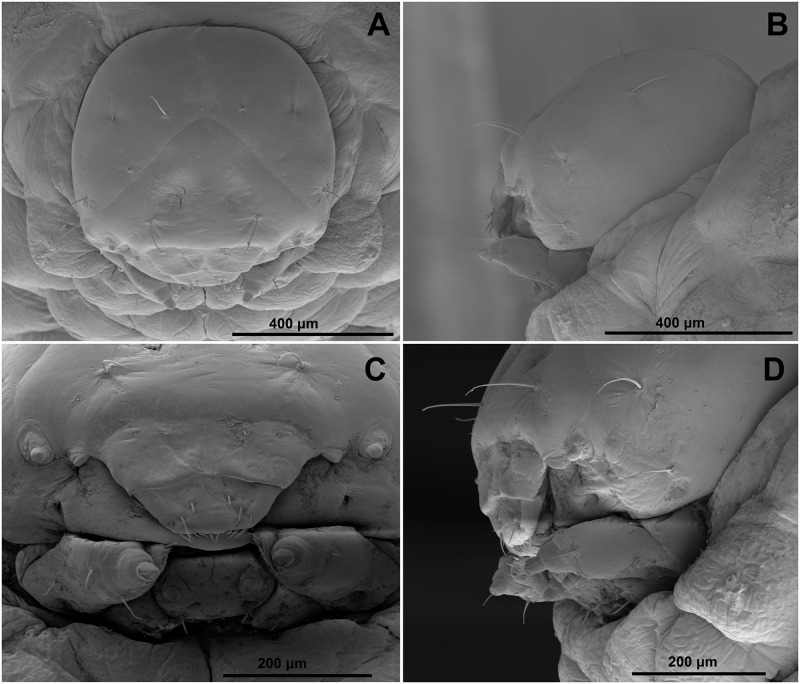
*Eucoeliodes mirabilis*: head and mouthparts of mature larva in SEM image. (A) Dorsal view. (B) Lateral view. (C) Dorsal view of clypeus and labrum. (D) Lateral view of mouthparts.

**Head capsule**: Head suboval, flattened laterally, endocarinal line absent (Figs 3A and 4A). Frontal sutures on head distinct, extended to antennae. Anterior and also posterior stemmata present, but feebly visible. *Des1* and *des2* located on upper part of the central part of epicranium, *des1* near the medial part of epicranium and *des2* near laterad of epicranium, *des3* located anteriorly near frontal suture, *des4* located in the central part of epicranium, and *des5* located anterolaterally; *des1*–*3* and *des5* relatively long, almost equal in length, *des4* very short to minute (Figs 3A and 4A). *Fs1* and *fs3* minute, placed medially, *fs2* absent, *fs4* relatively long, located anterolaterally, and *fs5* minute to very short, located laterally, close to the epistoma (Figs 3A and 4A). *Les* as long as *des1*; *ves1–2* very short (Fig 4B and 4D). Epicranial area with three setae (*pes1*–*3*) and 2 sensilla. Antennae located at the end of the frontal suture on each side, membranous and slightly convex basal article bearing conical, triangular sensorium, short; basal membranous article with 4 sensilla of different shape (Figs 3D, 4C and 4D). Clypeus (Figs 3E and 4C) approx. 2.5 times as wide as long, with 2 short *cls*, almost equal in length, located posterolaterally, and 1 sensillum located between *cls*; anterior margin concave.

**Mouthparts**: Labrum (Figs 3E and 4C) approximately 2.5–3 times as wide as long, with 3 pairs of piliform *lms* of different length; *lms3* very short, distinctly shorter than short *lms1* and *lms2*; *lms1* placed close to the margin with clypeus, *lms2* located anteromedially and *lms3* located anterolaterally; anterior margin double sinuate. Epipharynx (Fig 3F) with 3 blunt, slender, finger-like *als*, unequal in length; 2 very short, finger-like *ams*, *ams1* distinctly larger than *ams2*; 2 pairs of very short, blunt *mes*, unequal in length; labral rods (lr) elongated, sub-ellipse. Mandibles (Fig 3C) broad, trifid, tooth of unequal height; slightly truncate; both *mds* short, hairform, located in distinct alveoli. Maxilla (Fig 3B) stipes with 1 *stps*, 2 *pfs* and 1 *mbs*, *stps* and *pfs1*–*2* relatively long, almost equal in length, *mbs* very short; mala with 6 bacilliform *dms*; 5 short *vms*, distinctly different in length, 2 very short and 3 minute; *vms* distinctly shorter than *dms*. Maxillary palpi with two palpomeres; basal palpomere with 1 very short *mxps* and two sensilla; length ratio of basal and distal palpomeres: 1:0.9; distal palpomere with one sensillum and a group of conical, cuticular apical processes. Praelabium (Fig 3B) heart-shaped, with 1 short *prms*; ligula with sinuate margin and 1 piliform very short to minute *lig*; premental sclerite strongly sclerotized in ring-shaped with projections in the middle part (Fig 3B). Labial palpi with two palpomeres; length ratio of basal and distal palpomeres: 1:0.8; distal palpomere with one sensillum and short, cuticular apical processes; basal palpomere with 1 dorsal sensillum. Postlabium (Fig 3B) with 3 *pms*, *pms1* located anterolaterally, remaining two pairs more dorsolaterally; *pms1* and *pms3* very short to minute, *pms2* relatively long; surface of postlabium partly sclerotised.

**Fig 5:**
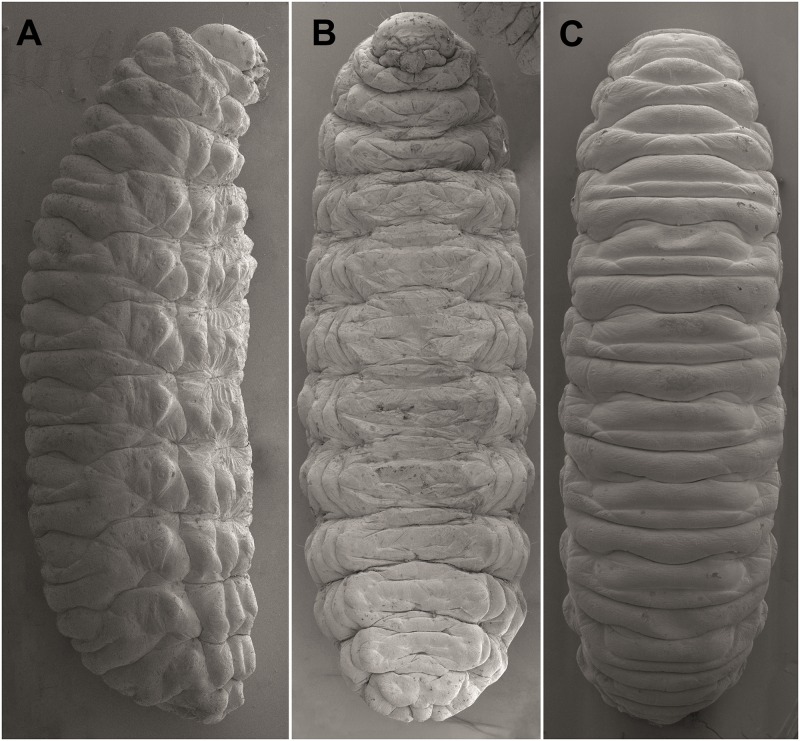
*Eucoeliodes mirabilis*: mature larva in a SEM image. (A) Lateral view. (B) Ventral view. (C) Dorsal view.

**Thorax and abdomen**: Body elongate, slightly curved, rounded in cross section (Fig 5A–5C). Prothorax distinctly smaller than meso- and metathorax. Abdominal segments I–V of almost equal length, next abdominal segments decreasing gradually to the terminal parts of the body. Abdominal segment X reduced to four anal lobes of unequal size; the dorsal lobe distinctly the largest, the lateral pair equal in size, and the ventral lobe very small. Anus located terminally. Spiracles (9 pairs) bicameral, the first placed between the pro- and mesothorax (see Materials and methods), the abdominal spiracles located laterally, close to the anterior margin of abdominal segments I–VIII; all of them have an elongated safety valve protected aperture against possible penetration of faeces inside (see in Chaetotaxy of mature larvae, and Discussion).

**Fig 6:**
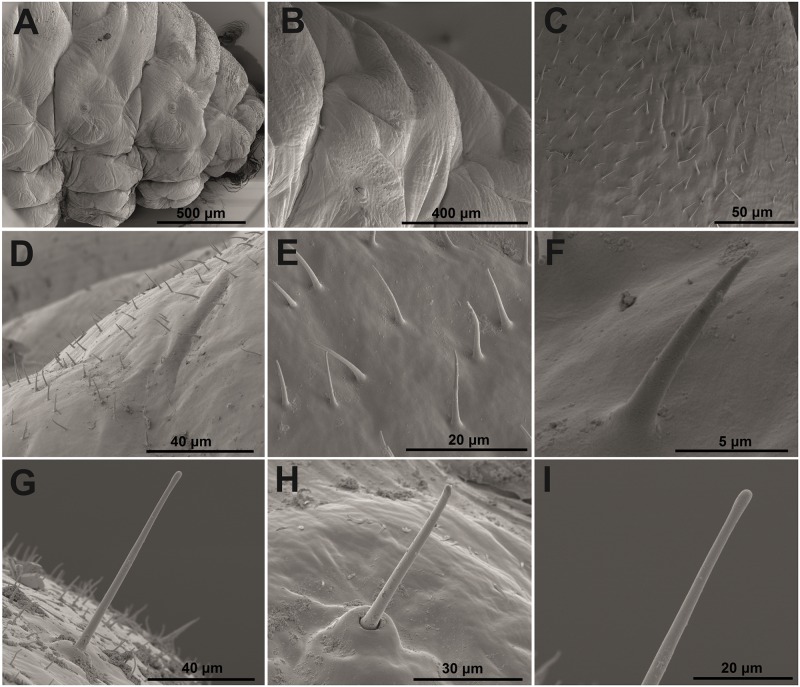
*Eucoeliodes mirabilis*: SEM photos of body with microtrichia and macroseta. (A) Lateral view of thorax at 500 μm. (B) Lateral view of thorax at 400 μm. (C) Microtrichiae at 50 μm. (D) Microtrichiae at 40 μm. (E) Microtrichiae at 20 μm. (F) Microtrichiae at 5 μm. (G) Seta at 40 μm. (H) Seta at 30 μm. (I) Apex of seta at 20 μm.

**Fig 7:**
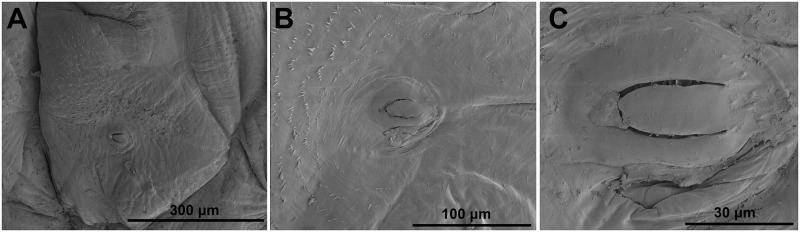
*Eucoeliodes mirabilis*: SEM photos of spiracles with microtrichia and valve. (A) Lateral view of spiracles at 300 μm. (B) Detail at 100 μm. (C) Detail at 30 μm.

**Chaetotaxy of mature larva**: Chaetotaxy of body very reduced, lower part completely without macrosetae. Setae thin, very short (up to minute) to sometimes short (one relatively long), light yellow or orange. Dorsal part of body up to spiracles covered by microtrichia (Fig 6A–6E, more in Discussion), with an approximate length of 10 μm, but variable. Density of microtrichia high, and space surrounding each was a maximum of ca 10 μm (see Fig 6A–6E). Microtrichia around spiracles distinctly stronger and in higher density, with some clusters (see Fig 7A–7C, more in Discussion).

**Fig 8:**
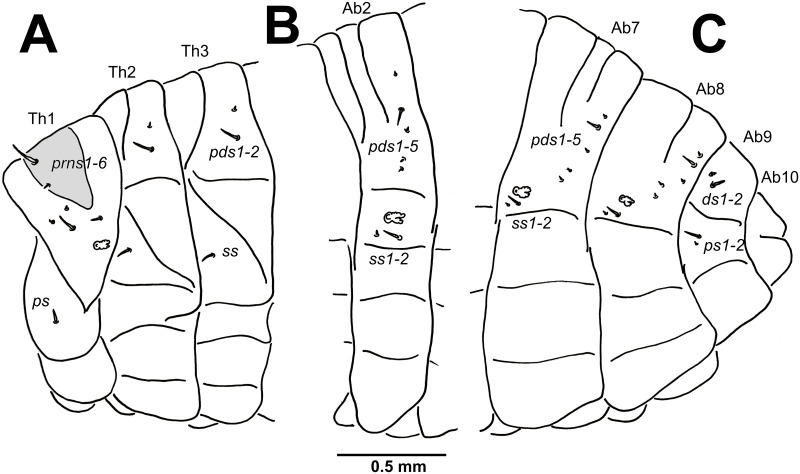
*Eucoeliodes mirabilis*: mature larva. (A) Lateral view of thoracic segments. (B) Lateral view of abdominal segment II. (C) Lateral view of abdominal segments VII–X. Abbreviations: Ab.–abdominal segment, Th.–thoracic s., I–X—number of segments, *prns*–pronotal seta(e), *pds*–postdorsal s., *ps*–pleural s., *ss*–spiracular s., and *ds*–dorsal s.

**Thorax**: Prothorax (Fig 8A) with 6 *prns* unequal in length (1 relatively long, 1 short, 1 very short and 3 minute), 2 on a weakly pigmented dorsal sclerite subdivided into two triangular plates medially, 4 close to spiracle; and 1 very short *ps*. Mesothorax (Fig 8A) with 2 *pds* unequal in length, *pds1* minute, *pds2* short; and 1 very short *ss*. Chaetotaxy of metathorax (Fig 8A) was identical to that of mesothoracal.

**Abdomen**: Abdominal segments I–VIII (Fig 8B and 8C) with 5 *pds*, *pds2* very short, remaining 4 setae minute; and 2 *ss* of unequal length, *ss1* minute, *ss2* very short. Abdominal segment IX (Fig 8C) with 2 *ds* (*ds1* minute, *ds2* very short); and 2 *ps* of unequal length, *ps1* very short, *ps2* minute. Abdominal segment X (Fig 8C) without setae.

#### Morphology of pupa

**Measurements (in mm)**: Body length: 3.9–4.3 (♂ 3.9–4.2; ♀ 4.0–4.3), at the widest region: 1.8–2.2. The widest place on the body is commonly between the apex of the meso- or metafemora.

**Colouration**: Body whitish to yellowish.

**Fig 9:**
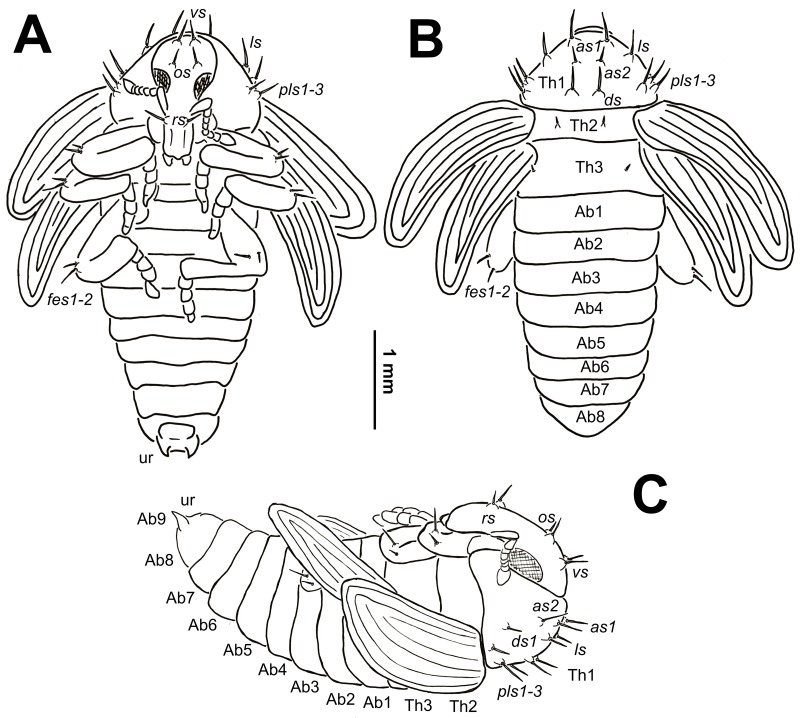
*Eucoeliodes mirabilis*: pupa habitus. (A) Ventral view. (B) Dorsal view. (C) Lateral view. Abbreviations: *vs*–vertical seta(e), *rs*–rostral s., *as*–apical s., *ls*–lateral s., *ds*–discal s., *pls*–posterolateral s., and *fes*–femoral s.; Th1-3 and Ab1-9–number of thoracic and abdominal segments, respectively, and ur—urogomphi.

**Morphology** (Fig 9A–9C): Body stocky. Cuticle smooth. Rostrum short and wide, approximately 2 times as long as wide, extended to mesocoxae. Antennae relatively long and stout. Pronotum from 1.6 to 1.8 times as wide as long. Mesonotum and metanotum of approximate equal length. Abdominal segments I–IV of almost equal length, abdominal segment V semicircular; next abdominal segments diminish gradually to the end of the body. Abdominal segments VI–IX distinctly smaller than the other abdominal segments. Gonotheca (abdominal segment IX) in females (2 specimens) bilobed. Sexual dimorphism in the weevils is visible primarily in the length of rostrum and in the structure of abdominal segment IX: gonotheca of ♂ undivided, of ♀ divided.

**Chaetotaxy** (Fig 9A–9C): Chaetotaxy of body very reduced. Setae relatively long on protuberances, unequal in length, light yellow or orange. Setae well visible. Head capsule included only 1 *vs* and 1 *os*. Rostrum with 1 *rs*. Setae on head capsule straight, as short as the remaining setae on thoracic and abdominal segments. Pronotum with 2 *as*, 1 *ds*, 1 *ls* and 3 *pls*, *as2* distinctly shorter than the remaining setae. Dorsal parts of meso- and metathorax with 1 seta located posterolaterally. Each apex of femora with groups of 2 *fes*. All abdominal segments I–VIII without setae. Urogomphi short, strong, triangular.

## Discussion

### Defensive strategies in ectophagous weevil larvae

Weevil larvae (Coleoptera: Curculionoidea) typically lead an endophytic life; however, some weevil groups live ectophytically [32]. Apart from the speciose subfamily Entiminae (more than 12 000 species in 1 370 genera) [40] with ectophytic but endophagous larvae (living in soil among roots), only a few species have both ectophytic and ectophagous larvae. Endophytic/endophagous larvae, either living in soil or inside plant tissues, are legless and largely sedentary, whereas ectophytic and ectophagous larvae live exposed on aerial parts of plants and have adaptations for moving on plant surfaces, including ambulatory ampullae, pedal lobes and creeping soles [32]. The ectophagous and ectophytic larvae are found in only a few groups, primarily most members of the subfamily Cyclominae [32, 41–43] and the unclassified tribes Bagoini, Gonipterini and Hyperini [32, 41, 43, 44]. These insects have all evolved strategies of physical or chemical defence against predators and parasitoids [45–48]. In Central Europe, in which this new defensive strategy in weevils was observed, ectophagous larvae, feeding on leaves or flowers, are currently found only in the following tribes: Bagoini, Cionini, Hyperini, Phytobiini and rarely Ceutorhynchini [32, 36, 49].

These known ectophagous larvae in Central Europe have three primary defensive strategies against predators and parasitoids. (1) The Hyperini are a well-known group of weevils with ectophagous larvae, which rely on cryptic coloration [45]. Members of the genus *Phelypera* from this tribe also benefit from the defensive strategy known as cycloalexy (a form of gregariousness that involves group reactions, see [46]). (2) Living in relatively unusual habitats is also a strategy to avoid predators and parasitoids because the pressure of parasitoids is partially decreased (e.g., Bagoini and Phytobiini live in aquatic habitats, and some Entiminae, e.g., *Otiorhynchus*, live in soil) [47]. The weevil tribe Cionini uses a different strategy (3) in which viscid mucus covers and protects larval bodies [48], but this mucus is primarily protection from desiccation [50].

Larvae of the subfamily Ceutorhynchinae live primarily endophytically [51], and the only known exception is *Ranunculiphilus faeculentus* (Gyllenhal, 1837), with ectophytic larvae that feed on terminal buds of *Consolida regalis* Grey (Ranunculaceae) [52]. Our biological investigations showed that all larval stages of *E*. *mirabilis*, which is also in the tribe Ceutorhynchini, feeds ectophytically on leaves and used a faecal shield for covering the entire dorsum. The shield is obviously green when squeezed and is most likely of vegetal origin. We hypothesized that the shield played a defensive role but also prevented desiccation.

To date, very little is known about the biology of the weevil *E*. *mirabilis*, which is also the case for species of the closely related genus *Coeliodes* Schoenherr, 1837. The life history is well documented only for another close relative, *Pseudocoeliodes rubricus* (Gyllenhall, 1837), in which the larvae develops in male flowers of *Pistacia* [53]. This species is univoltine and overwinters as an adult. In spring, adults feed on host plants. Larvae develop in male flowers and floral axes and pupate in soil at the end of April/beginning of May. Adults of the new generation aestivate after a short feeding [53]. Based on this information, many previous authors reached the obvious conclusion that larval development in flowers or flower buds is likely for the genera *Coeliodes*, *Coeliodinus* Dieckmann, 1972 and *Eucoeliodes* (all Coleoptera: Curculionidae: Ceutorhynchini) [27, 30, 49], although this type of development has never been supported by direct observation, including in our field study. Larval feeding marks of *E*. *mirabilis* closely resembled those of *Steronychus fraxini* (DeGeer, 1775) (Coleoptera: Curculioninae: Cionini), which feeds on the leaves of *Fraxinus* (R. Stejskal, pers. observ.).

### Morphological adaptations to fix the faecal shield in beetles

Currently, faecal ecology in beetles is known only for the immature stages of leaf beetles. Within Chrysomelidae, three primary defensive strategies use faeces with morphological adaptations: 1, super-anal processes to carry defensive shields composed of shed larval skins (exuviae) and faeces that are often retained after pupation (Cassidinae); 2, portable enclosures of bell-shaped cases of faeces and also plant material (Lamprosomatinae and Cryptocephalinae); and 3, accumulation of faeces on the dorsum to partially or entirely cover the larvae (Criocerinae and Blepharida-group Galerucinae).

Using faeces as a defensive shield in *E*. *mirabilis* is a strategy similar to that in the Criocerinae. The faecal shield in this group is a physical and also probable chemical barrier against predators and parasitoids, but compared with the Criocerinae, different morphological adaptations have developed in *E*. *mirabilis*. Only a few Criocerinae larvae [18, 54] are well described, and the knowledge about the variability of their morphological characters is very poor. Anyway, it is known that the dorsal vestiture of a few known Criocerinae larvae is covered by short, relatively sparse setae [18, 54] unlike that of *E*. *mirabilis*, whose macrosetae are completely reduced and the dorsal vestiture is covered with a high density of microtrichia (Fig 6A–6E). Based on the character and arrangement, these microtrichia likely act effectively as a velcro. Because of the high density of microtrichia on the dorsal vesture, the faecal shield is most likely removed only during moulting, in contrast to the Criocerinae, which have short and sparse setae on the dorsum [18] and the faecal shield is easily removed by unfavourable conditions.

Moreover, distinct morphological adaptations for protecting spiracles against faeces have also been found on *E*. *mirabilis* larvae. The density of microtrichia around all spiracles was high, and the microtrichia are in clusters and are slightly shorter and distinctly stronger. These morphological differences all contributed to the protection from and the prevention of faecal incursion into the tracheal system. However, the microtrichia is not the only morphological adaptation, and all spiracles are also protected by a safety valve, which reduces the space in which faeces might penetrate. Spiracles of Criocerinae larvae are minute but distinct [54], unlike those of *E*. *mirabilis*, which are protected by the altered microtrichia and safety valve (Fig 6A–6E). These morphological adaptations, including velcro on the dorsal vesture, the system of protection around spiracles and the safety valve in spiracles, are all unique, and these adaptations were used in combination to use faeces as a defensive strategy in *E*. *mirabilis*.

### Comparison with immature stages of other Ceutorhynchinae species

The larvae of 58 Ceutorhynchinae taxa in 22 genera have been described previously [36, 51, 55–65]. A detailed description of the pupa is known for only six Ceutorhynchinae taxa [36, 56, 62, 63]. The immature stages were compared with most of the species described or drawn by Anderson [56, 57], Scherf [36], May [59], Lee and Morimoto [60], Orlova-Bienkowskaya [61], Gosik [62–64] and Nikulina [65]; these illustrations are all of high or sufficient quality and therefore were very useful; however, the described characteristics are useful only for differential diagnosis. The comparison of the larvae and pupae with some species described by Scherf [36] was somewhat difficult due to the use of different terminology for morphology and chaetotaxy and/or the absence of good-quality drawings and data about some important morphological features. In that case, the comparison with such taxa has not been possible, and they cannot be included in the *Key to the immatures of the subfamily Ceutorhynchinae*.

The most precise general description of larvae of the subfamily Ceutorhynchinae, which is summarized by 9 character sets, is presented by May [59]: (1) head with *des3* on epicranial half (Fig 3A); (2) antennae hemispherical, fully exposed (Fig 3A and 3D); (3) endocarinal line absent (Fig 3A); (4) frons only with *fs4* developed (Fig 3A); (5) tormae separate, subparallel (Fig 3F); (6) postlabium with proximal pairs of *plbs* as far apart as the median pair (Fig 3B); (7) abdominal spiracles with air-tubes caudad (Fig 8B and 8C); (8) abdominal VIII spiracle lateral (Fig 8C); and finally (9) abdominal segments with ventral lobes developed as ambulatory ampullae (Fig 5A–5C). Almost all these characters fit with known descriptions [36, 51, 55–65]. There is observed only one exception, the statement number 4 –frons only with *fs4* developed. The larvae of *E*. *mirabilis* has frons with long *fs4*, but is has also three other short to minute setae (*fs1*, *fs3* and *fs5*) (see Fig 3A). This feature is not consistent with other descriptions, e.g., by Anderson [57] (*Ceutorhynchus rapae* Gyllenhal, 1837; larvae have short, but distinct *fs5*), Orlova-Bienkowskaya [61] (*Phytobius leucogaster* (Marsham, 1802) (published as *Litodactylus lecuogaster*) with 6 *fs*), Gosik [63] (*Tapeinotus sellatus* (Fabricius, 1794) with 3 *fs*), Gosik [64] (*Mogulones austriacus* (Ch. Brisout de Barneville, 1869) and *M*. *dimidiatus* (Frivaldszky, 1865) has 4 *fs*), and Nikulina [65] (*C*. *subtilirostris* Schultze, 1902 and *C*. *viator* Faust, 1885; larvae have short, but distinct *fs1*).

Macrosetae on larval and also pupal bodies of *E*. *mirabilis* are completely reduced (Figs 8A–8C and 9A–9C). All described morphological adaptations in *E*. *mirabilis*, including velcro on the dorsal vesture, the system of protection around spiracles and the safety valve in spiracles (more in previous chapter), are absolutely unique not only in the subfamily Ceutorhynchinae, but also completely in all weevils and probable also in all beetles. Larvae of *E*. *mirabilis* have also some other unique characters in the subfamily Ceutorhynchinae as follows; (1) trifid mandible (Fig 3C), and (2) only two *ams* and also two *mes* on epipharynx (Fig 3F). All known larvae of Ceutrohynchinae have only bifid mandibles, and three *ams* and two *mes* on epipharynx. The position of absent *ams* seta is often problematic. According to Marvaldi [38, 39], the standard status of the epipharynx in weevils is 2 *ams* and 3 *mes*, but when the position of the distal *mes* is very close to the anterior margin, they appear as *ams*. The decision was finally made to add this problematic seta to the latter group (*ams*), and the position of this seta is similar to that in other genera, e.g., in *Coniocleonus* Motschulsky, 1860 or *Tychius* Germar, 1817. We did not follow Stejskal et al. [66] and Skuhrovec et al. [67], who accepted the standard status in weevils and counted the seta as *mes*, but we followed Trnka et al. [68] and Skuhrovec et al. [69], e.g., in *Adosomus* Faust, 1904 or *Sibinia* Germar, 1817. Count of setae on ventral side of maxilla (*vms*) is also different from other species, but this is not a unique feature, and in this case, there are probably many mistakes in descriptions as for example descriptions of two *Poophagus* species [62], where probably 2 *vms* are counted as *dms* in *Poophagus hopffgarteni* Tournier, 1873.

### Key to the immatures of the subfamily Ceutorhynchinae

#### Larvae

The following key is based on a recent description of immature stages in the genus *Eucoeliodes* and descriptions of immature stages published before: *Ceutorhynchus* (8 species) [36, 55, 57, 60, 65]; *Eubrychius* (1 species) [61]; *Glocianus* (1 species) [36]; *Homorsoma* (1 species) [60]; *Hypurus* (1 species) [60]; *Mecysmoderes* (1 species) [60]; *Mogulones* (5 species) [58, 64]; *Phrydiuchus* (2 species) [56]; *Phytobius* (1 species) [61]; *Poophagus* (2 species) [62]; *Rhinoncus* (2 species) [59, 60]; *Tapeinotus* (1 species) [63]; and *Trichosirocales* (1 species) [59]. Unfortunately, some basal data about chaetotaxy on body is missing, and then it is not possible to distinguish all genera (see accumulation of genera in point 6). Further comments are reported in the previous chapter in Discussion.

1. Head broadly and deeply emarginate at posterior margin. ………………………… ***Hypurus***

-. Head normally rounded posteriorly. ………………………………………………………… **2**

2. Eighth abdominal spiracles on papillae. Air-tubes of spiracles different in length, dorsal air-tube about half as long as ventral air-tube. …………………….……………… ***Mecysmoderes***

-. Eighth abdominal spiracles not on papillae, but near dorsal bases of papillae or in normal position. ……………………………………………………………………………………… **3**

3. Dorsal part of body covered by vestitutre with many microtrichia. …………… ***Euceoliodes***

-. Dorsal part of body covered by vestitutre without any microtrichia. ……………………… **4**

4. Frons with 5 or more *fs*. ………………………………….……………….……… ***Phytobius***

-. Frons with 4 or less *fs*. …………………………………….………………………………… **5**

5. Dorsal epicranium with 5 *des*. …………………………………………………… ***Tapeinotus***

-. Dorsal epicranium with less than 5 *des*. ……………………………………………………… **6**

6. Dorsal epicranium with 3 *des*. …………………………………………….………………… **7**

-. Dorsal epicranium with 4 *des*. …………………………………………………….………………… ***Ceutorhynchus*, *Homorsoma*, *Mogulones*, *Phrydiuchus*, *Poophagus*, *Trichosirocales***

7. Abdominal segments I–VII with 4 *pds*. ………………………………………… ***Rhinoncus***

-. Abdominal segments I–VII with less than 4 *pds*. …………………………………………… **8**

8. Abdominal segments I–VII with 3 *pds*. ………………………………………… ***Eubrychius***

-. Abdominal segments I–VII with 1 *pds*. …………………………………………… ***Glocianus***

#### Pupae

The following key is based on recent description of pupa in the genus *Eucoeliodes* and descriptions of pupae published before: *Glocianus* (1 species) [36]; *Phrydiuchus* (2 species) [56]; *Poophagus* (2 species) [62]; and *Tapeinotus* (1 species) [63].

**1**. Rostrum elongated, at least 5 times as long as wide. ………………………………………… **2**

-. Rostrum short and wide, about 2 times as long as wide. …………………………. ***Eucoeliodes***

**2**. *Pas* present. ………………….……………………………………………………………… **3**

-. *Pas* absent. …………………………………………………………………………………… **4**

**3**. Meso- and metathorax and also abdominal segments without seta. Femora with 1 seta.…….…………………………………………………………………….…………………. ***Tapeinotus***

-. Meso- and metathorax with 1 seta, and abdominal segments with more than 3 setae on dorsal part. Femora with 2 setae. ……………………………………………….…. ***Phrydiuchus***

**4**. Rostrum very slender, 8 times as long as wide, *sos* absent. ……….……………. ***Poophagus***

-. Rostrum elongated, 5 times as long as wide, *sos* present. …………………………. ***Glocianus***

## Conclusions

Our observations confirm that the larvae of *E*. *mirabilis* feed exclusively ectophytically on leaves. The appearance of *E*. *mirabilis* larvae on leaves is remarkable, with the protective faecal shield covering the entire body. Although most similar to the strategy adapted by the Criocerinae, the defensive shield as a strategy is unique in *E*. *mirabilis*, and the larvae have clearly different morphological adaptations. Macrosetae on larval and also pupal bodies of *E*. *mirabilis* are completely reduced, and the vesture is covered with a high density of microtrichia, which serve as velcro. Because of the high density of microtrichia on the dorsal vesture, the faecal shield is most likely removed only during moulting. As a system to protect spiracles against incursion of faeces into the tracheal system, the density of microtrichia around all spiracles is high, and the microtrichia are slightly shorter, distinctly stronger, and arranged in clusters. All spiracles are also protected by a safety valve, which decreases the space in which faeces could enter. All these mentioned morphological adaptations are unique and used in combination to use faeces as a defensive strategy in *E*. *mirabilis*. Finally, the generic keys for identification of larvae and pupae based on useful preliminary descriptions is also presented. All these new data about biology and also morphology of *E*. *mirabilis* are very useful in next studies of immature stages in Polyphaga beetles, but there are also still some missing gaps (e.g. chemical composition of host plant tissues, and feces; description of shield construction; morphology of younger instars, etc.).
